# Bibliometric analysis of global research trends between gut microbiota and breast cancer: from 2013 to 2023

**DOI:** 10.3389/fmicb.2024.1393422

**Published:** 2024-07-31

**Authors:** Xianguang Deng, Hua Yang, Lingjia Tian, Jie Ling, Hui Ruan, Anqi Ge, Lifang Liu, Hongqiao Fan

**Affiliations:** ^1^Department of Galactophore, The First Hospital of Hunan University of Chinese Medicine, Changsha, Hunan, China; ^2^Department of Cosmetic and Plastic Surgery, The First Hospital of Hunan University of Chinese Medicine, Changsha, Hunan, China

**Keywords:** gut microbiota, breast cancer, bibliometrics, probiotics, metabolomics

## Abstract

**Background:**

Breast cancer is the most prevalent cancer globally and is associated with significant mortality. Recent research has provided crucial insights into the role of gut microbiota in the onset and progression of breast cancer, confirming its impact on the disease’s management. Despite numerous studies exploring this relationship, there is a lack of comprehensive bibliometric analyses to outline the field’s current state and emerging trends. This study aims to fill that gap by analyzing key research directions and identifying emerging hotspots.

**Method:**

Publications from 2013 to 2023 were retrieved from the Web of Science Core Collection database. The VOSviewer, R language and SCImago Graphica software were utilized to analyze and visualize the volume of publications, countries/regions, institutions, authors, and keywords in this field.

**Results:**

A total of 515 publications were included in this study. The journal *Cancers* was identified as the most prolific, contributing 21 papers. The United States and China were the leading contributors to this field. The University of Alabama at Birmingham was the most productive institution. Peter Bai published the most papers, while James J. Goedert was the most cited author. Analysis of highly cited literature and keyword clustering confirmed a close relationship between gut microbiota and breast cancer. Keywords such as “metabolomics” and “probiotics” have been prominently highlighted in the keyword analysis, indicating future research hotspots in exploring the interaction between metabolites in the breast cancer microenvironment and gut microbiota. Additionally, these keywords suggest significant interest in the therapeutic potential of probiotics for breast cancer treatment.

**Conclusion:**

Research on the relationship between gut microbiota and breast cancer is expanding. Attention should be focused on understanding the mechanisms of their interaction, particularly the metabolite-microbiota-breast cancer crosstalk. These insights have the potential to advance prevention, diagnosis, and treatment strategies for breast cancer. This bibliometric study provides a comprehensive assessment of the current state and future trends of research in this field, offering valuable perspectives for future studies on gut microbiota and breast cancer.

## Graphical abstract

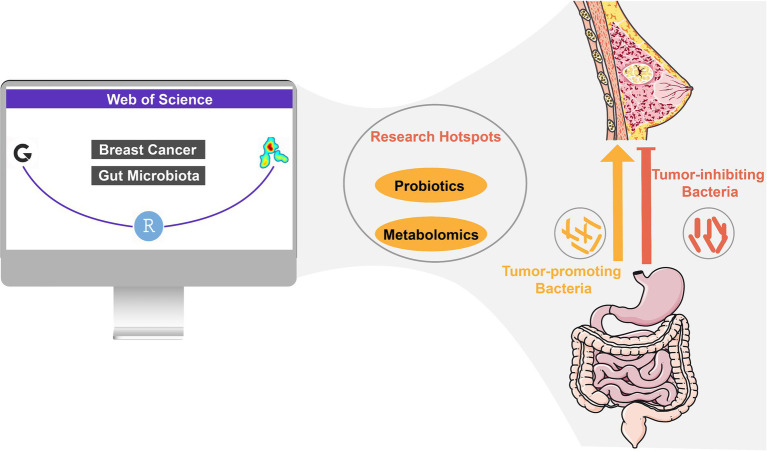

## 1 Introduction

Breast cancer is currently recognized as the most prevalent malignant neoplasm globally, accompanied by a relatively high mortality rate (Sung et al., 2021). Moreover, there is an alarming trend toward a younger demographic increasingly being diagnosed with this disease (Bidoli et al., 2019). It not only presents substantial health risks but also imposes considerable psychological burdens on those afflicted with breast cancer. For many years, research on the pathogenesis of breast cancer predominantly focused on genetic and environmental factors (Swaminathan et al., 2023). However, these factors did not sufficiently explain the underlying causes of breast cancer. With the advancement of bacterial DNA sequencing technologies in recent years, numerous studies have discovered a close correlation between gut microbiota and the development of various cancers, including breast cancer (Papakonstantinou et al., 2022). This emerging evidence underscores the potential role of gut microbiota in influencing breast cancer pathogenesis (He et al., 2021), thereby expanding our understanding of its etiology beyond traditional genetic and environmental perspectives.

The human gastrointestinal tract is home to a complex ecological system comprising thousands of bacteria, fungi, viruses, and other microorganisms (Álvarez et al., 2021). The dynamic composition of this gut microbiota is subject to influence and regulation by a multitude of factors. These factors include the host’s diet, hormonal levels, medication use, and environmental conditions, all of which can significantly impact the quantity and diversity of microbes present in the gastrointestinal tract. Alterations in certain endogenous or exogenous factors can lead to microbial dysbiosis, which, in turn, may contribute to the development of a variety of diseases (Wargo, 2020). This intricate relationship underscores the critical role of gut microbiota in maintaining overall health and the potential implications of its imbalance in disease pathogenesis.

Over the past decade, numerous studies have linked gut microbiota to various diseases, including cardiovascular diseases such as atherosclerosis and coronary heart disease (Trøseid et al., 2020; Rahman et al., 2022), respiratory diseases such as asthma and chronic obstructive pulmonary disease (Barcik et al., 2020; Wei et al., 2023), autoimmune diseases such as inflammatory bowel disease (Sugihara and Kamada, 2021) and rheumatoid arthritis (Zhao et al., 2022), and various malignant tumors (Xie et al., 2022). An increasing body of research has demonstrated a significant correlation between gut microbiota and the development and progression of breast cancer (Alpuim Costa et al., 2021; Álvarez-Mercado et al., 2023). Numerous studies have confirmed that certain gut microorganisms influence the prognosis of breast cancer patients (Urbaniak et al., 2014; Xuan et al., 2014; Zhang et al., 2023). For example, *Blautia* sp. has been found to have a higher abundance in higher-grade breast cancer tissues, and it can promote the development of breast cancer (Wu A. H. et al., 2022). Concurrently, therapeutic approaches targeting alterations in gut microbiota were developed for the treatment of breast cancer (Mendoza, 2019). Specifically, *Akkermansia muciniphila* has been proven to inhibit the occurrence and development of breast cancer by enhancing the body’s immune capabilities. Currently, attempts are being made to treat breast cancer by adjusting the abundance of *Akkermansia muciniphila* (Bernardo et al., 2023). This field of study has evolved significantly over time, with an increasing number of publications underscoring the pivotal role of gut microbiota in the pathogenesis of breast cancer and opening new avenues for innovative therapeutic strategies. However, there is currently a lack of comprehensive analysis of these published studies.

Bibliometrics is a statistical methodology that employs public literature databases for the analysis and visualization of key elements within research fields, such as keywords, authors, countries, and the volume of publications (Hu et al., 2022). This approach has been extensively utilized to analyze developmental trends and research hotspots within specific areas of study (Wang et al., 2021). Numerous studies have employed bibliometric analysis to explore the relationship between gut microbiota and various cancers, including colorectal cancer (Wu H. et al., 2022) and lung cancer (Chen et al., 2023). With the incidence of breast cancer increasing in recent years, there has been a surge in literature examining the relationship between gut microbiota and breast cancer. However, no study to date has utilized bibliometric analysis to investigate this specific link. Therefore, in this study, we undertook a bibliometric analysis of relevant literature from the past decade within the Web of Science database, specifically focusing on the research area of gut microbiota and breast cancer. This analysis was intended to provide researchers with a comprehensive understanding of the state of research at that time and to facilitate a better grasp of future research directions in this field.

## 2 Methods

### 2.1 Data sources and search strategies

**Figure 1:**
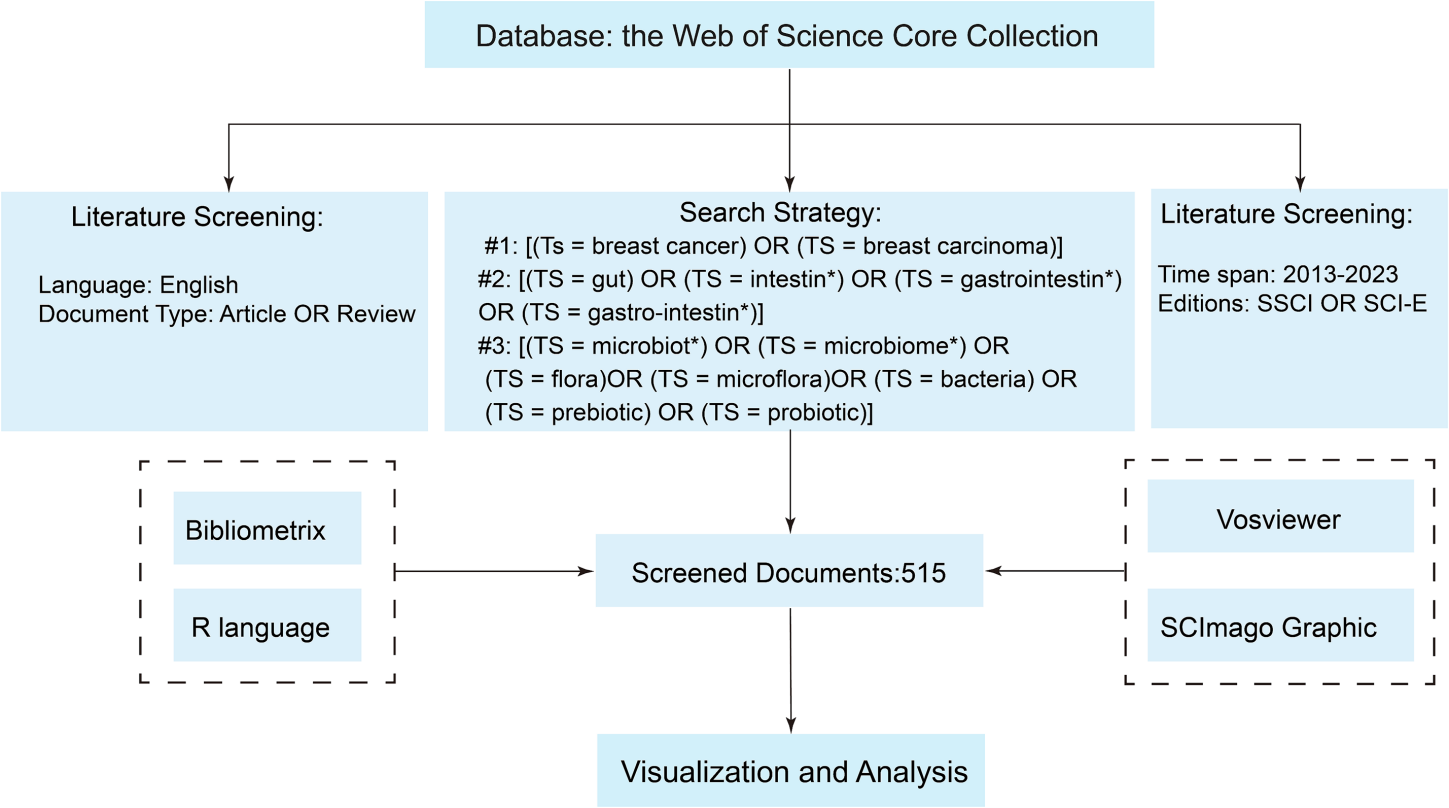
The flowchart of the literature selection process in this study.

The original data used in this study were downloaded from the Web of Science Core Collection. We conducted our search based on topic search (Ts) and used the asterisk “*” as a wildcard to ensure a comprehensive search that included all relevant variants of the term. The specific search strategy we employed was as follows: #1: [(Ts = breast cancer) OR (TS = breast carcinoma)]; #2: [(TS = gut) OR (TS = intestin*) OR (TS = gastrointestin*) OR (TS = gastro-intestin*)]; #3: [(TS = microbiota*) OR (TS = microbiome*) OR (TS = flora) OR (TS = microflora) OR (TS = bacteria) OR (TS = prebiotic) OR (TS = probiotic)]. The final dataset was the intersection of #1 AND #2 AND #3. The search spanned from 1 January 2013 to 31 December 2023. Publications were restricted to those in English. The data recorded included complete records and cited references, with the file format being plain text. To minimize information loss, we did not set specific exclusion criteria. Instead, we employed a manual screening method to exclude irrelevant literature. The detailed criteria and process for literature selection in this study were illustrated in Figure 1. All the original data used in this study were derived from publicly available databases. Therefore, an ethical review was not required.

### 2.2 Data analysis

The Bibliometrix R package (version 4.1.3; Aria and Cuccurullo, 2017) was used to analyze publication trends, journal distribution, and highly cited publications. VOSviewer (version 1.6.19; Eck and Waltman, 2009), in conjunction with SCImago Graphica, was employed to visually analyze the collaboration and distribution of countries/regions, institutions, and authors. In our institutional analysis, we included all institutions listed by the corresponding authors to ensure comprehensive information retention and to align with the capabilities of existing bibliometric software. Keyword clustering was visually analyzed using VOSviewer. For the analysis of keyword temporal distribution, data on keywords were exported using the Bibliometrix R package, and heatmaps of keyword distribution over time were created using R software.

## 3 Results

### 3.1 General analysis

**Figure 2:**
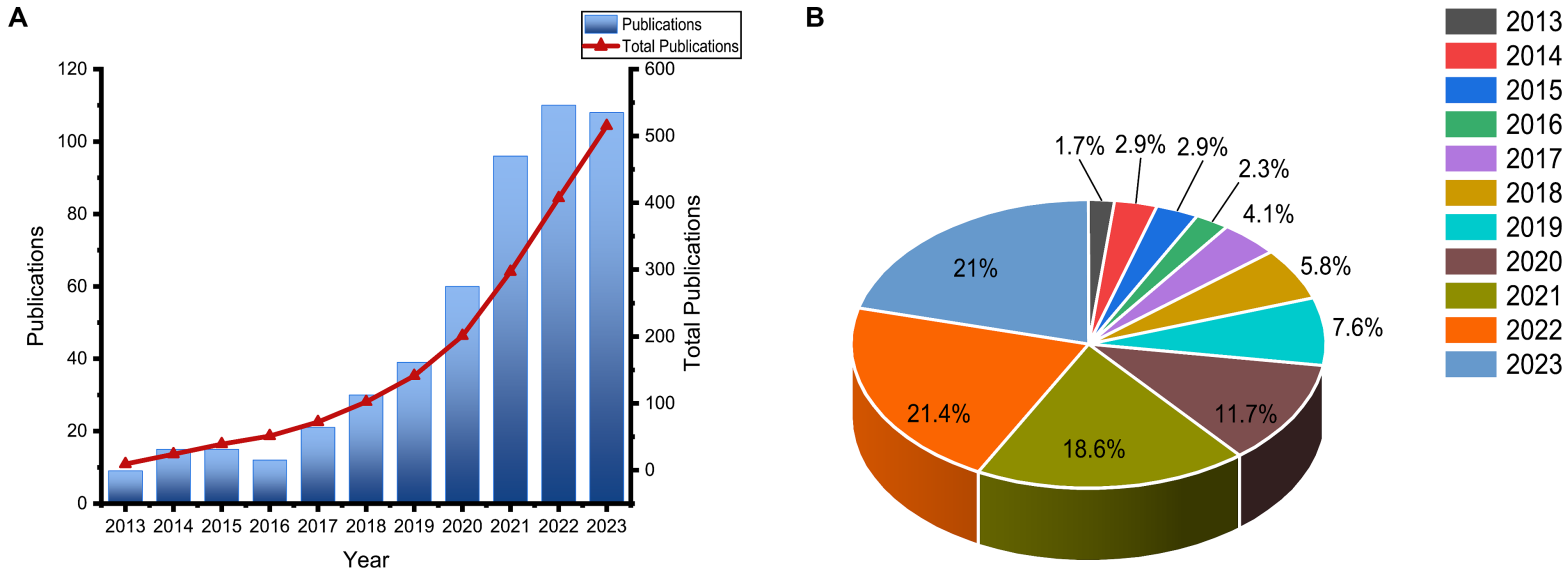
From 2013 to 2023, over the past decade, the trend in the number of articles published related to gut microbiota and breast cancer was observed. **(A)** The annual and cumulative publications on gut microbiota and breast cancer; **(B)** The proportion of the number of articles each year within the total number of articles.

In this study, a total of 515 publications that investigated the relationship between the gut microbiota and breast cancer were included. Among these, 291 were research articles and 224 were review papers. Figure 2 displayed the publication trend in the research field of gut microbiota and breast cancer. Overall, prior to 2018, there was a modest amount of research on the relationship between gut microbiota and breast cancer. Until 2018, the quantity of publications began to increase steadily, with the number of publications in the last 4 years accounting for 60% of the total publications over the past decade.

### 3.2 Analysis of countries/regions

**Figure 3:**
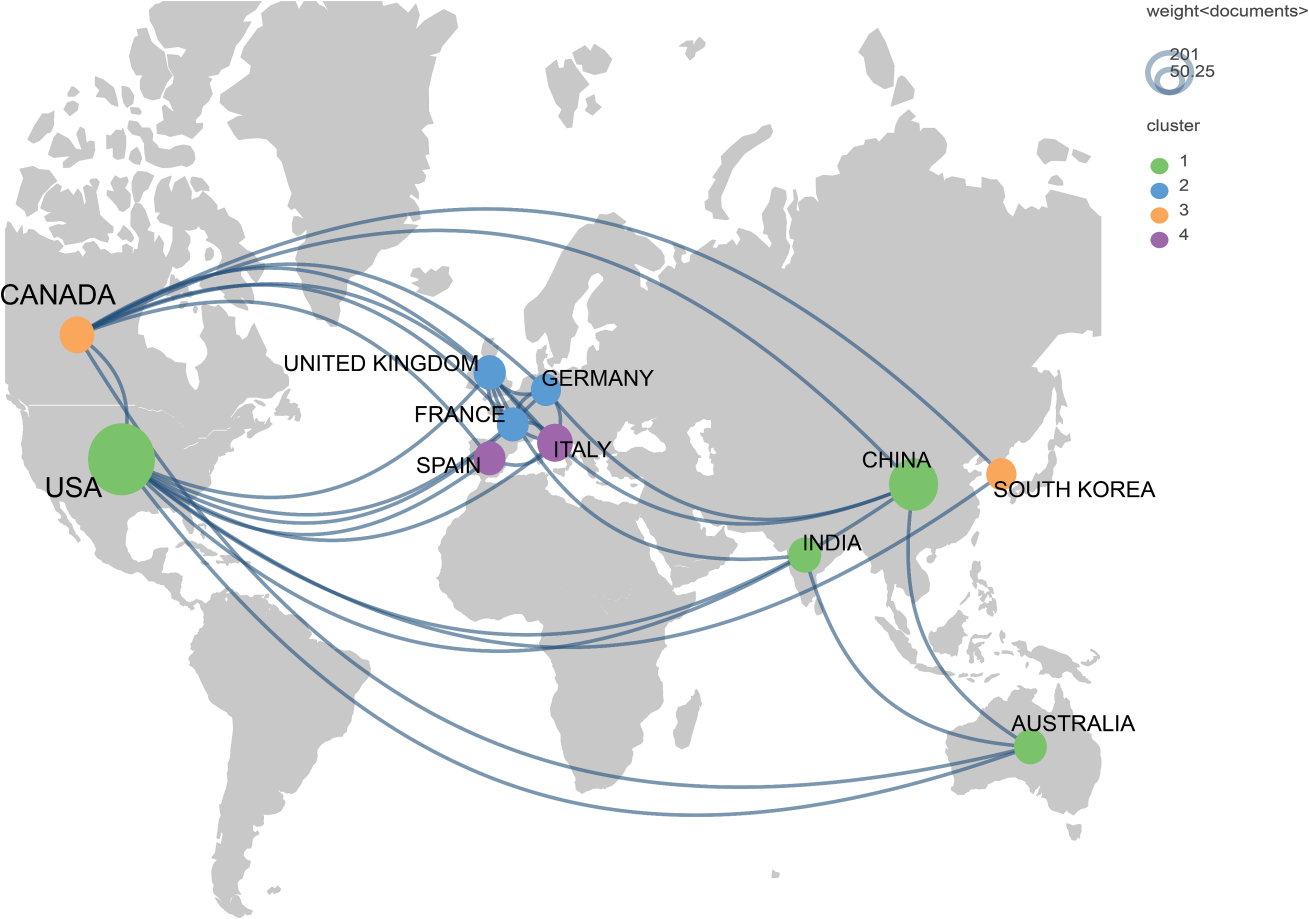
Geographic bibliometric map based on a network of co-authorship relationships in the top 11 countries in terms of number of articles published.

Over the past decade, 64 countries/regions participated in research on breast cancer and gut microbiota. In terms of publication volume, the United States had the highest number of publications, followed by China, Italy, Australia, and Canada. In terms of citation counts, the United States was the country with the most citations, followed by Italy, Canada, and France (Table 1). The cooperative network among these countries was illustrated in Figure 3, which showed that the countries displayed each had no fewer than 15 publications in this research field, totaling 11 countries within this network map. From the diagram, it was evident that the United States and Canada had more collaborations with other countries.

**Table 1:** The top 11 countries in terms of the number of publications.

Rank	Country	Publications	Total citations	Average citation
1	USA	201	9,063	45.09
2	China	92	1,482	16.11
3	Italy	33	2,375	71.97
4	Canada	29	1,355	46.72
5	Australia	23	570	24.78
6	India	23	227	9.87
7	United Kingdom	22	484	22
8	France	19	777	40.89
9	Spain	19	620	32.63
10	Germany	15	393	26.2
11	South Korea	15	151	10.07

### 3.3 Contribution of institutions

**Figure 4:**
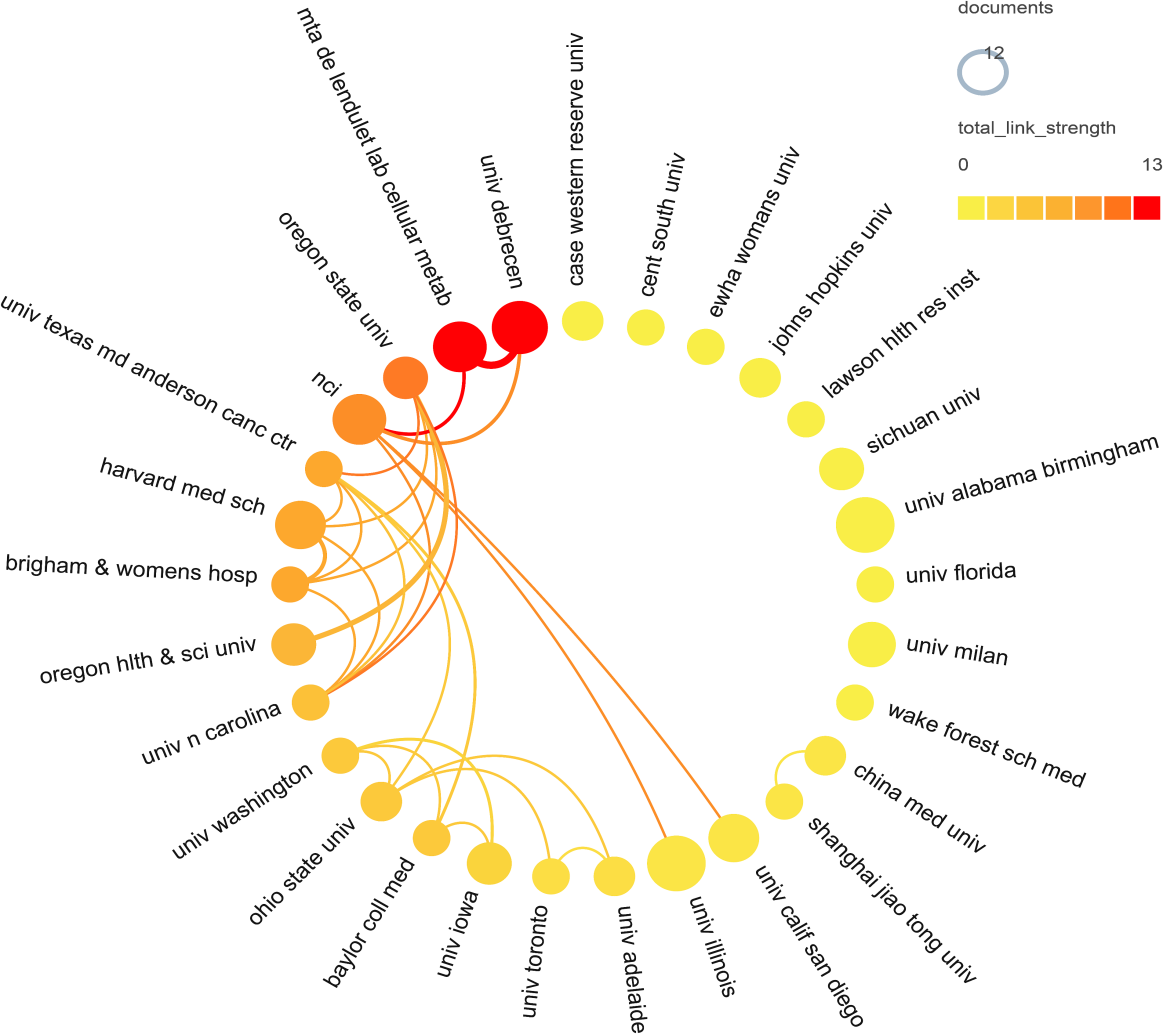
Institutional cooperation network map. In the network, number of documents reflected by node size. The connection strength is reflected by the color.

A total of 1,012 institutions contributed to the research on breast cancer and gut microbiota. We assessed the most productive institutions, as shown in Table 2, the University of Alabama Birmingham had the highest number of contributions with 12 papers, followed by the University of Illinois and the University of Debrecen. In terms of citation counts, the University of California, San Diego, received the highest number of citations, despite having published only 9 papers. Following were the National Cancer Institute with 809 citations, the University of Debrecen with 636 citations, and the MTA DE Lendület Laboratory of Cellular Metabolism with 571 citations. The institutional collaboration cluster network was depicted in Figure 4, displaying 29 institutions that all had more than 5 publications each. Among these, The University of Debrecen and the MTA DE Lendület Laboratory of Cellular Metabolism maintained a strong cooperative relationship. The Oregon State University engaged in collaborations with multiple institutions. However, other institutions had fewer academic collaborations in this field.

**Table 2:** The top 12 institutes in terms of the number of publications.

Rank	Institute	Publications	Citations	Average citation
1	University of Alabama Birmingham	12	218	18.17
2	University of Illinois	12	224	18.67
3	University of Debrecen	11	636	57.82
4	MTA DE Lendület Laboratory of Cellular Metabolism	10	571	57.1
5	National Cancer Institute	10	809	80.9
6	Harvard Medical School	9	175	19.44
7	University of California, San Diego	9	1,081	120.11
8	University of Milan	8	207	25.88
9	Oregon Health and Science University	7	0	0
10	Oregon State University	7	16	2.29
11	Sichuan University	7	171	24.43
12	University of Iowa	7	34	4.86

### 3.4 Analysis of authors

**Figure 5:**
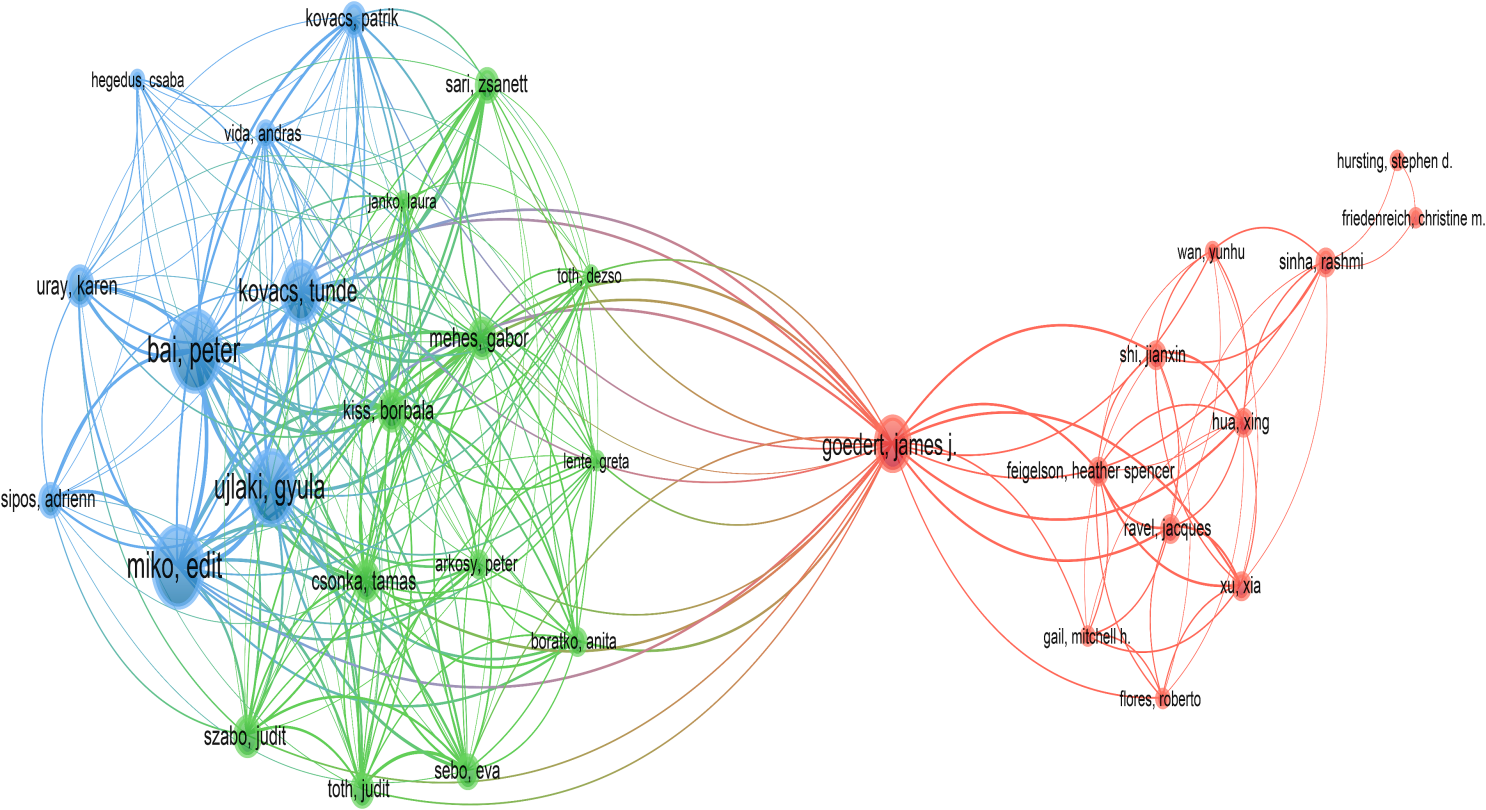
The collaboration network diagram within the field was depicted. Nodes represented authors, with the size of the points indicating the volume of publications. Lines between points signified collaborations among authors.

A total of 3,052 distinct authors contributed to the 515 publications included in this study. Table 3 displayed the top 10 authors by publication volume, with each author having published at least 6 related articles. Bai, Peter ranked first, followed by Miko, Edit, and Ujlaki, Gyula. In terms of citation counts, Goedert, James J received the highest number of citations, followed by Bai, Peter, and Miko, Edit. Figure 5 illustrated the network of collaborations among authors. Cluster analysis revealed that three main collaborative clusters had formed, with Goedert, James J. occupying a central position and collaborating with the other two academic groups.

**Table 3:** The top 5 authors in terms of number of publications.

Rank	Author	Publications	Citations	Average citation
1	Bai, Peter	11	636	57.82
2	Miko, Edit	11	636	57.82
3	Ujlaki, Gyula	10	580	58
4	Cook, Katherine L	8	171	21.38
5	Kovacs, Tunde	8	457	57.13
6	Morrow, Casey D.	8	211	26.38
7	Goedert, James J	7	767	109.57
8	Chun, Brie	6	0	0
9	Karstens, Lisa	6	0	0
10	Parida, Sheetal	6	185	30.83

### 3.5 Analysis of journals

**Figure 6:**
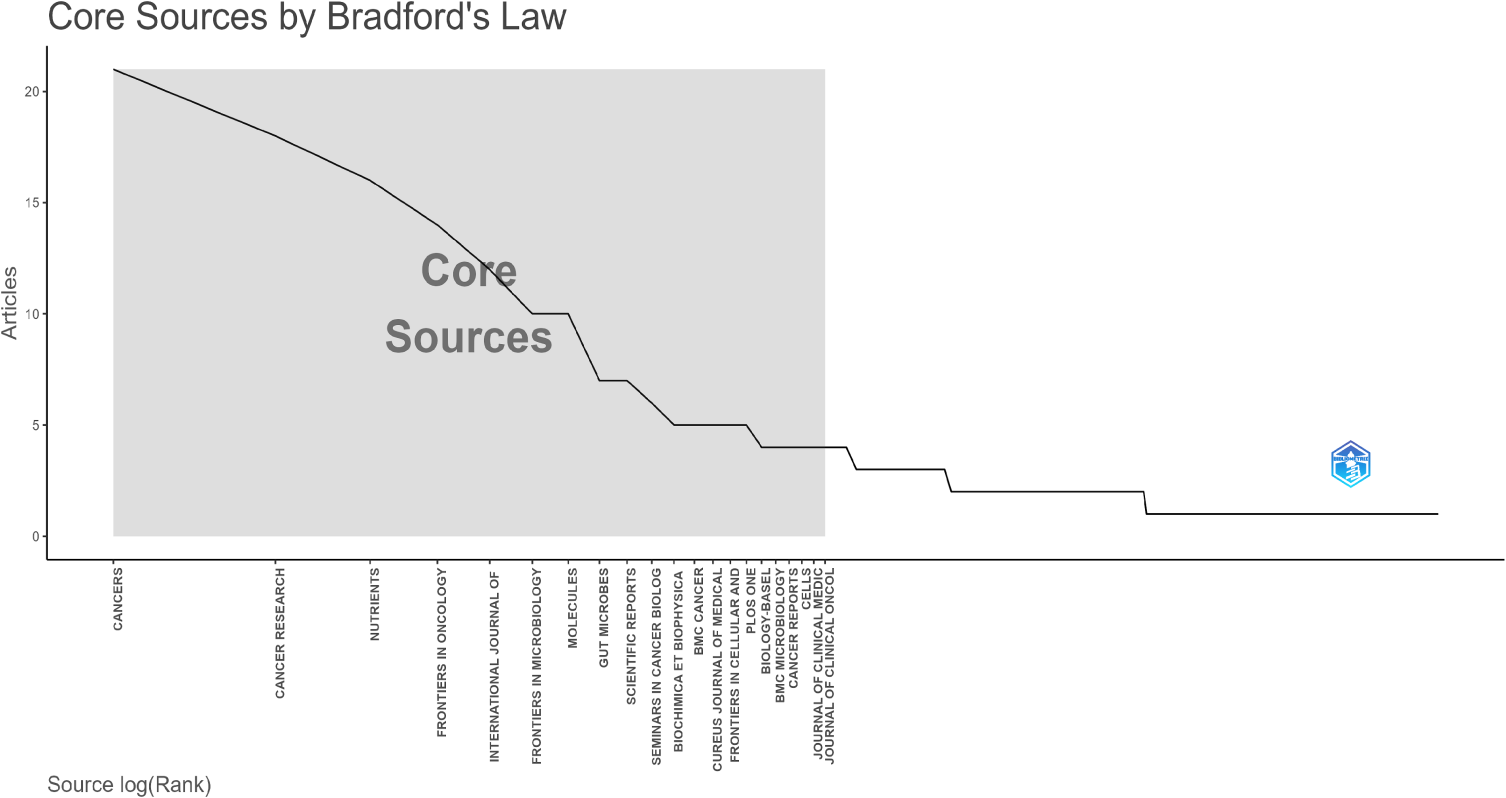
The distribution map of core journals was illustrated. According to Bradford’s Law, one-third of the articles in this field were located in the core source zone, encompassing a total of 21 journals.

All selected articles were published across 289 journals. Table 4 listed the top 10 most prolific journals. The journal with the highest number of publications was *Cancers*, followed by *Cancer Research* and *Nutrients*. The impact factors of these journals all exceeded 4, with *Seminars in Cancer Biology* having the highest impact factor. In terms of citation counts, *Seminars in Cancer Biology* received the highest number of citations (384), followed by *Nutrients* (347) and *Gut Microbes* (313). Furthermore, as illustrated in Figure 6, we applied Bradford’s Law to identify the core source journals for research on the relationship between gut microbiota and breast cancer, determining 21 core journals.

**Table 4:** The top 10 leading journals.

Rank	Journal	Total articles	Total citations	Average citations	IF(2023)
1	Cancers	21	384	18.29	5.2
2	Cancer Research	18	220	12.22	11.2
3	Nutrients	16	347	21.69	5.9
4	Frontiers in Oncology	14	178	12.71	4.7
5	International Journal of Molecular Sciences	12	78	6.5	5.6
6	Frontiers in Microbiology	10	80	8	5.2
7	Molecules	10	170	17	4.6
8	Gut Microbes	7	313	44.71	12.2
9	Scientific Reports	7	155	22.14	4.6
10	Seminars in Cancer Biology	6	136	22.67	14.5

### 3.6 High-cited articles

Highly cited literature is considered an important evaluation metric and often represents research with significant impact in the field. Bibliometrix introduces two concepts: “Global Citation Score (GCS)” and “Local Citation Score (LCS).” GCS refers to the total number of times an article is cited in the Web of Science, while LCS refers to the number of citations within all the literature we included.

The selected literature encompassed a total of 34,981 references. Table 5 displayed the top 10 highly cited documents based on local citations. The article by Goedert, J. J. et al., titled “Investigation of the association between the fecal microbiota and breast cancer in postmenopausal women: a population-based case–control pilot study” published in the *Journal Of The National Cancer Institute*, received the highest number of local citations (98) and also had a significant GCS (221). The document with the highest GCS was authored by Urbaniak, Camilla et al., titled “Microbiota of human breast tissue” published in *Applied and Environmental Microbiology*, which achieved the highest GCS (310).

**Table 5:** The top 10 most local cited documents.

Author	Title	Year	Journal	Local citations	Global citations	DOI
Goedert, J. J et al.	Investigation of the association between the fecal microbiota and breast cancer in postmenopausal women: a population-based case–control pilot study	2015	Journal of the National Cancer Institute	98	221	10.1093/jnci/djv147
Urbaniak Camilla et al.	The Microbiota of Breast Tissue and Its Association with Breast Cancer	2016	Applied and Environmental Microbiology	92	328	10.1128/AEM.01235-16
Xuan Caiyun et al.	Microbial dysbiosis is associated with human breast cancer	2014	PLoS One	82	304	10.1371/journal.pone.0083744
Zhu Jia et al.	Breast cancer in postmenopausal women is associated with an altered gut metagenome	2018	Microbiome	72	141	10.1186/s40168-018-0515-3
Fernández Mariana F et al.	Breast Cancer and Its Relationship with the Microbiota	2018	International Journal of Environmental Research and Public Health	72	174	10.3390/ijerph15081747
Kwa Maryann et al.	The Intestinal Microbiome and Estrogen Receptor-Positive Female Breast Cancer	2016	Journal of the National Cancer Institute	64	280	10.1093/jnci/djw029
Urbaniak Camilla et al.	Microbiota of human breast tissue	2014	Applied and Environmental Microbiology	63	310	10.1128/AEM.00242-14
Fuhrman Barbara J et al.	Associations of the fecal microbiome with urinary estrogens and estrogen metabolites in postmenopausal women	2014	The journal of Clinical Endocrinology and Metabolism	55	187	10.1210/jc.2014-2222
Mikó Edit et al.	Microbiome-Microbial Metabolome-Cancer Cell Interactions in Breast Cancer-Familiar, but Unexplored	2019	Cells	53	109	10.3390/cells8040293
Luu Trang H et al.	Intestinal Proportion of *Blautia* sp. is Associated with Clinical Stage and Histoprognostic Grade in Patients with Early-Stage Breast Cancer	2017	Nutrition and Cancer	46	96	10.1080/01635581.2017.1263750

### 3.7 Keywords co-occurrence network

**Figure 7:**
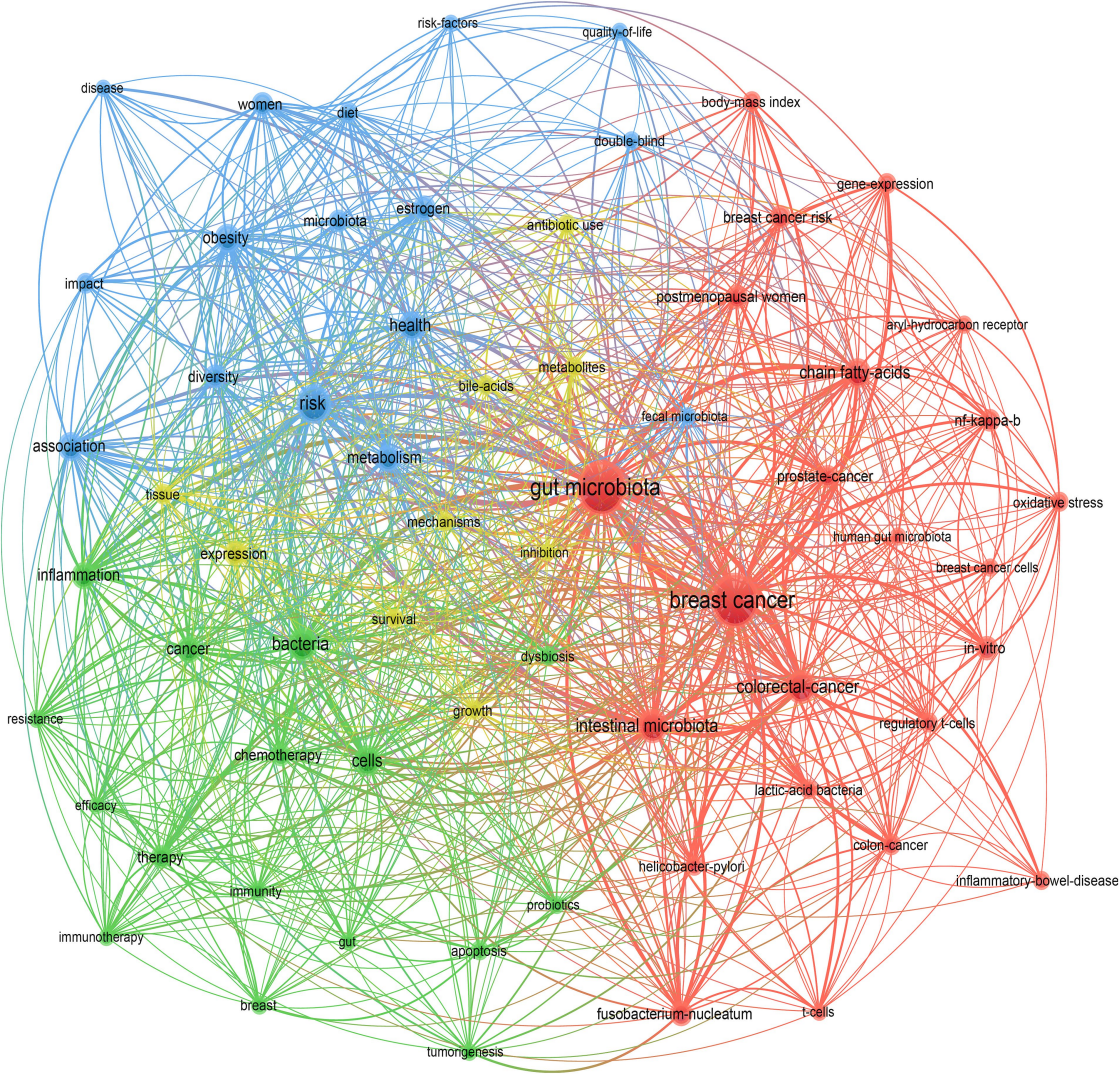
The keyword co-occurrence network clustering diagram.

The analysis of keyword co-occurrence provided a comprehensive summary of the relationships between keywords. By examining the co-occurrence network, we were able to understand the research hotspots and trends. The keyword co-occurrence map, as shown in Figure 7, considered the esthetics and readability of the graphic, keywords that appeared a minimum of 10 times in these articles were included, totaling 64 keywords for this study. Each keyword was represented by a node, with closely related keywords grouped into a cluster representing a significant direction within the core research area. Nodes of the same color belonged to the same cluster. In our analysis, 4 clusters were identified. These clusters had overlapping and intersecting characteristics, indicating that research in this field was neither dispersed nor isolated.

The red cluster contained 23 nodes, with key terms mainly including “breast cancer,” “gut microbiota,” “postmenopausal women,” “breast cancer risk,” “lactic-acid bacteria,” “intestinal microbiota,” “*Helicobacter pylori*,” etc. This cluster focused on the commonly addressed theme in the field, primarily exploring the association between gut microbiota and breast cancer and demonstrating several types of gut microorganisms related to the development of breast cancer, such as lactic-acid bacteria and *Helicobacter pylori*.

The green cluster included 16 nodes, with keywords like “bacteria,” “chemotherapy,” “resistance,” “therapy,” “immunotherapy,” etc. The cluster primarily involved the relationship between the gut microbiota and treatments related to breast cancer, indicating that the gut microbiome influenced the effectiveness of breast cancer treatment.

The blue cluster comprised 16 nodes, with keywords such as “diet,” “microbiota,” “association,” “metabolism,” “estrogen,” risk” etc. These keywords were closely related to metabolism, and these metabolic factors were also highly relevant to the development and progression of breast cancer. This indicated that these metabolic factors influenced changes in the gut microbiota, participating in the onset process of breast cancer.

The yellow cluster contained 9 nodes, with key terms including “bile acids,” “metabolites,” “survival,” “inhibition,” “growth,” etc. This cluster indicated that metabolites, such as bile acids, affected the gut microbiota, thereby influencing the survival of breast cancer patients.

**Figure 8:**
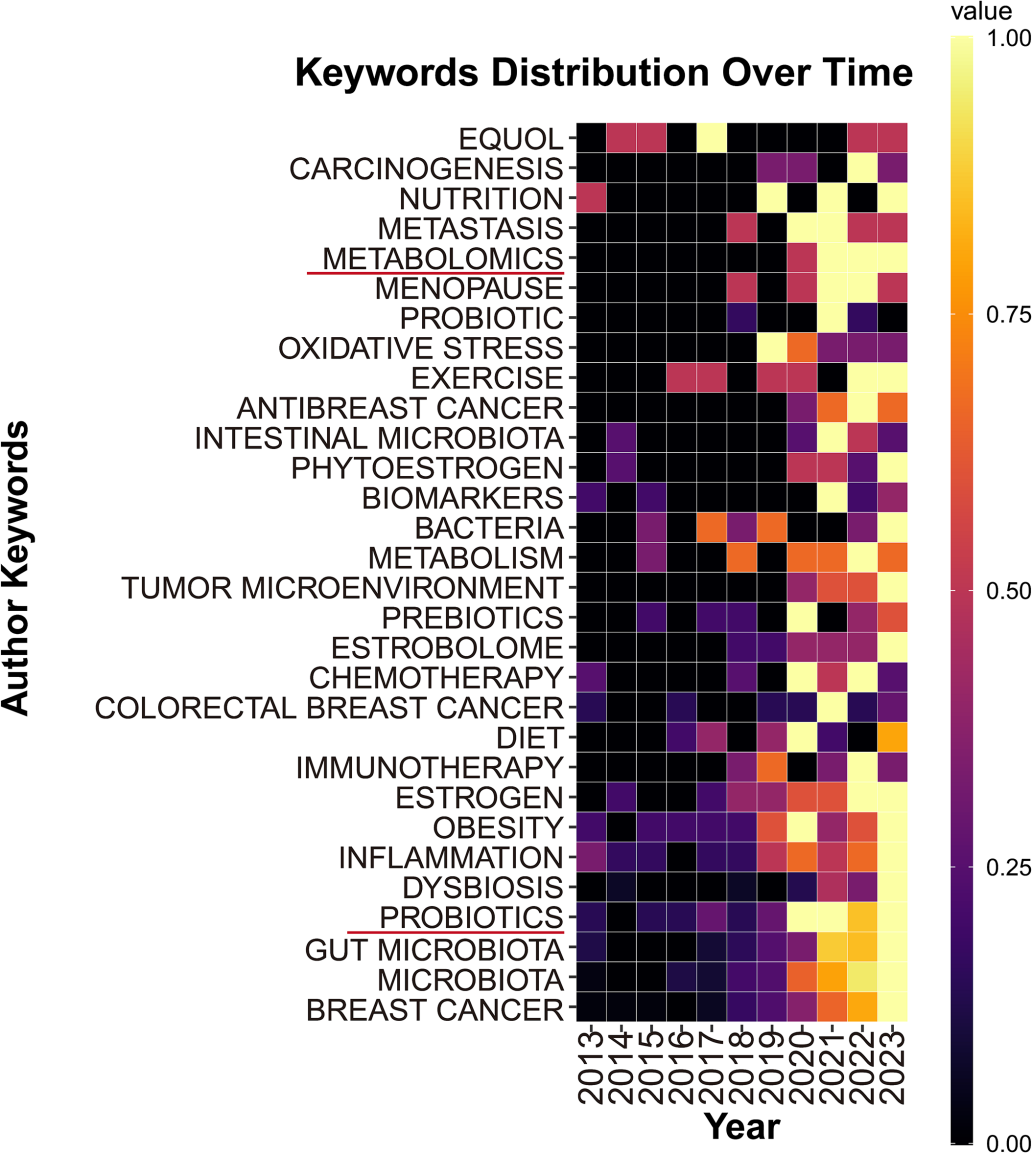
The keywords distribution over time.

To further explore the changes in research topic hotspots, we also used high-frequency keywords (keywords appearing more than 30 times) to create a heatmap of keyword distribution over time. As shown in Figure 8, keywords such as “metabolomics” and “probiotics” had higher occurrence frequencies.

## 4 Discussion

### 4.1 Trends in publications

Numerous studies indicated that dysbiosis of gut microbiota is closely related to the development and progression of breast cancer, leading to an increasing number of researchers engaging in the study of the relationship between gut microbiota and breast cancer. However, a bibliometric analysis of this field has not been conducted until now. In this study, by screening 515 eligible research articles, we analyzed the publication volume, countries, institutions, authors, journals, highly cited documents, and keywords within this field. As observed in Figure 2, the year 2018 marked a significant turning point for research in the area of gut microbiota and breast cancer, with a rapid increase in the number of publications until 2018. This surge could be attributed to the maturation and widespread adoption of sequencing technologies, and the publication volume in the last 3 years remained around 100 articles per year, indicating that research on the relationship between gut microbiota and breast cancer is currently a hot topic.

### 4.2 International collaboration

The United States was the leading country in terms of publication volume, with a total of 201 articles, significantly surpassing China’s 92 articles, and Italy’s publication volume was 59 articles less than China’s. The publication outputs of the top three countries exhibited considerable disparities. This phenomenon underscored the dominant position of the United States and China in this research field, potentially due to the higher incidence rates of breast cancer and the larger number of researchers engaged in this field of study in the two countries. Notably, not only was the United States’ publication volume high, but its average citation count was also significant, suggesting the high quality of its articles. Although Italy had a lower publication volume, it ranked first in terms of average citation count, indicating the impactful nature of its published articles.

In the institutional analysis, we found that the most productive institutions were predominantly located in the United States. While there was not a significant difference in publication volume among the institutions, there was a notable disparity in citation counts. It is worth mentioning that the University of California, San Diego, despite having published only 9 articles, accumulated a total of 1,091 citations, averaging 120.11 citations per article, indicating its significant influence in this field. However, when looking at the collaboration network diagrams among countries (Figure 3), institutions (Figure 4), and authors (Figure 5), the cooperation was not very close, which could potentially limit the advancement of scholarship in this area. Therefore, for further academic development, we strongly suggested enhancing academic collaboration between authors from different countries and institutions to foster scholarly progress. Additionally, the research success of these institutions relates to the diverse group of researchers with expertise in this field and the substantial financial funding available to them. This high research productivity is expected, considering it occurs within institutions that have stronger infrastructure, richer scientific resources, and extensive experience in microbiome research.

According to the journal analysis, the top 10 journals published 23.5% of the articles. These journals were primarily focused on oncology, microbiology, nutrition and metabolism, and chemistry. Among them, oncology journals published the most articles, indicating that gut microbiota has become a focal point in breast cancer research.

### 4.3 Research hotspots and Frontiers

Currently, various cancers have been extensively studied in the context of gut microbiota, with bibliometric methods revealing research hotspots and future trends (Fernandes et al., 2022). This is one of the most crucial functions of bibliometric analysis. For example, Wu W. et al. (2022) identified “*F. nucleatum*” and “probiotics” as key focus areas and trends in gut microbiota research related to colorectal cancer through bibliometric analysis. Similarly, Wu S. et al. (2023) highlighted that future research hotspots in the gut microbiota of pancreatic cancer will focus on microbiota-based diagnostics and treatments. Thus, bibliometric analysis can help scholars identify future research directions and trends. An analysis of the top 10 most-cited articles revealed that research mainly concentrated on three themes: (1) alterations in the gut microbiota of breast cancer patients, with a particular focus on postmenopausal women, likely considering the impact of estrogen on breast cancer (Goedert et al., 2015). The studies found differences in the abundance of 45 types of microorganisms between postmenopausal patients and healthy postmenopausal individuals, including *Escherichia coli*, *Shewanella putrefaciens* and *Erwinia amylovora*, among other microorganisms (Zhu et al., 2018). (2) The microbiome and its relationship with the development and progression of breast cancer (Kwa et al., 2016). Estrogen levels are closely linked to breast health, and studies found that the gut microbiome regulated the body’s serum estrogen levels (Mikó et al., 2019), thereby affecting the development and progression of breast cancer. Therefore, regulating those microorganisms that influence estrogen levels might be an important strategy for treating breast cancer. (3) The impact of specific microorganisms on breast cancer. Various gut microorganisms were found to affect the development and progression of breast cancer. For instance, studies discovered that the intestinal proportion of *Blautia* sp. was associated with the clinical staging and histological grade of prognosis in early-stage breast cancer patients (Luu et al., 2017). These studies have preliminarily revealed the interactions between breast cancer, gut microbiota, and breast cancer-related factors. However, the current understanding of how specific gut microorganisms influence particular aspects of breast cancer remains unclear and requires further investigation.

Based on our keyword co-occurrence analysis, it was discernible that breast cancer is closely associated with changes in gut microbiota. Through clustering analysis, the red cluster led to the inference that many gut microorganisms are risk factors for breast cancer. From Figure 7 we observed that the microorganisms closely associated with breast cancer were lactic-acid bacteria and *Helicobacter pylori*. It has been reported that lactic-acid bacteria possess anti-carcinogenic properties against various cancers, including breast cancer (Wu et al., 2021). Studies found that lactic-acid bacteria exhibited anti-proliferative effects on breast cancer cells (Swacita et al., 2022), reduced tumor size, and possessed significant anti-angiogenic and anti-metastatic properties (Ranjbar et al., 2019; Shi et al., 2023), as well as inhibited breast cancer stem cells (Choi et al., 2018). Currently, researchers consider it as a potential adjuvant in the treatment of breast cancer (Levit et al., 2021). Moreover, there was no direct evidence to suggest that *Helicobacter pylori* was directly related to the development and progression of breast cancer. However, *Helicobacter pylori* induces a state of chronic inflammation in the body (Li, 2023) and is closely related to the body’s estrogen receptor levels (Kang et al., 2022). Therefore, *Helicobacter pylori* might establish a link with breast cancer through inflammation and by elevating estrogen receptor levels. An experimental study discovered that using recombinant *Helicobacter pylori* proteins altered the rate of cytokine production and enhanced the tumoricidal activity of the immune system, thereby treating breast cancer (Mohabati Mobarez et al., 2020). Hence, regulating the gut microbiome might become an important direction for the future treatment of breast cancer.

Through the green clustering in Figure 7, it became evident that the gut microbiome had a close relationship with the treatment of breast cancer, including chemotherapy, resistance to drugs, and immunotherapy (Laborda-Illanes et al., 2020). These treatments not only impacted the gut microbiome but the gut microbiome also played a role in modulating the treatment outcomes for breast cancer (Plaza-Díaz et al., 2019). Studies revealed that the gut microbiome of breast cancer patients underwent significant changes following chemotherapy treatment. Specifically, the relative abundance of the mucin-degrading genus *Akkermansia* significantly decreased (Wu R. et al., 2022). Furthermore, the alterations in the gut microbiome caused by chemotherapy were closely linked with cognitive impairments and symptoms of depression observed after the treatment (Bilenduke et al., 2022). Another study highlighted that the microbiome was associated with prognostic and immunological characteristics of breast cancer, such as histological subtypes, cancer staging, receptor status, and lymph node positivity. The study identified that propionibacteria and staphylococci exhibited a negative correlation with carcinogenic immunological features (De Silva et al., 2021; Tzeng et al., 2021). Moreover, the gut microbiome influenced the resistance of breast cancer to treatment (Viswanathan et al., 2023) and the effectiveness of trastuzumab therapy (Di Modica et al., 2021). Therefore, treatments related to breast cancer altered the richness of the gut microbiome, which in turn could influence the therapeutic efficacy of breast cancer treatment.

The blue cluster primarily depicted the association between dysbiosis of the gut microbiota and risk factors related to breast cancer. It was evident that considerable research focused on the link between diet, estrogen, obesity, and other breast cancer factors with the gut microbiome. The structure of the diet was closely related to the incidence of breast cancer (De Cicco et al., 2019; González-Palacios Torres et al., 2023). In a case–control study, through 16 s rRNA sequencing, it was found that the fecal samples of breast cancer patients with lower intake of vegetables, dairy products, and fruits showed a significant increase in the microorganisms Acidaminococcus, Tyzzerella, and Hungatella. This indicated that dietary structure affected the gut microbiome of breast cancer patients, and the gut microbiota, in turn, served as a biomarker for assessing the risk of breast cancer (Papadimitriou et al., 2021; Altinok Dindar et al., 2023). High estrogen levels are found to promote the development and progression of breast cancer (Wen et al., 2017). Studies revealed that gut microorganisms expressing the enzyme β-glucuronidase could increase the bioavailability of estrogen, thereby leading to the occurrence of breast cancer (Arnone and Cook, 2022). Consequently, inhibitors of enzyme β-glucuronidase were considered potential therapeutic drugs for breast cancer (Ervin et al., 2019). Several gut microbiota species related to estrogen were identified, and current research also involves interventions through diet or medication to alter the levels of estrogen receptors, aiming to treat breast cancer or enhance the efficacy of endocrine therapy (Heath et al., 2024). Obesity was recognized as a significant risk factor for the occurrence and recurrence of breast cancer (Picon-Ruiz et al., 2017), and it was also closely associated with dysbiosis of the gut microbiome (Liu et al., 2021). Studies found that obesity reduced the α-diversity of the gut microbiome (Hossain et al., 2021) and that obesity-related pathogenic gut microorganisms regulated premetastatic niches, thereby promoting lung and liver metastases of breast cancer (Fu et al., 2022; Parida et al., 2023). Furthermore, not only did research confirm that regulating obesity-related gut microorganisms could treat breast cancer, but it could also prevent the occurrence of obesity-related breast cancer (Chen et al., 2022). Therefore, risk factors for breast cancer were closely related to the gut microbiome, and reducing the recurrence risk of breast cancer through modulation of the gut microbiota emerged as an important research focus.

From the yellow cluster, it was inferred that the gut microbiome could even affect the survival of breast cancer patients. Bile acids, closely related to the gut microbiome, play a critical role in the digestion and absorption of fats and vitamins (Collins et al., 2023). Studies revealed that breast cancer patients with high bile acid metabolism had higher survival rates and lower histological grades. Conversely, patients with low bile acid metabolism exhibited a significant increase in gut microorganisms such as *Lactobacillus*, *Ruegeria*, and *Marichromatium*, which were highly associated with the proliferation of breast cancer cells, thereby promoting tumor proliferation and growth. Therefore, breast cancer patients with low bile acid metabolism might have lower survival rates (Wu R. et al., 2022). Additionally, *Fusobacterium nucleatum* was found to inhibit the accumulation of infiltrating T cells in invasive tumors, thereby reducing the Disease-Free Survival of breast cancer patients. Surprisingly, the use of antibiotics could reverse this phenomenon (Parhi et al., 2020; Simin et al., 2020). These studies demonstrated that in-depth investigation into the role of the gut microbiome in breast cancer held significant importance for improving the survival rates of breast cancer patients.

As shown in Figure 8, from the keyword temporal distribution chart, “metabolomics” and “probiotics” appeared with high frequency in recent years, indicating that the two keywords might become sustained hot topics in the future. Metabolomics technology has become increasingly mature and received widespread promotion and application. Metabolomics was extensively applied to investigate various metabolic dysregulations in breast cancer (Mukherjee et al., 2022), especially in terms of the interplay between glucose metabolism and gut microbiota (Al-Ansari et al., 2021). Apart from having a unique approach to glucose metabolism (Farhadi et al., 2022), breast cancer also exhibited different lipid and protein energy metabolism compared to normal cells (Gandhi and Das, 2019; Farhadi et al., 2022), which had been less studied up to that point, thus offering significant room for further research. Therefore, employing metabolomics to investigate the interactions between various metabolites and the gut microbiome may become a sustained research direction in the coming years. The impact of probiotics on cancer has become a prominent research topic (Zyoud et al., 2022). Our study also revealed that utilizing probiotics to modulate gut microbiota for breast cancer treatment has emerged as a significant area of interest. Probiotics competed with pathogens for adhesion sites on the intestinal mucosa and nutrients, which helped maintain the natural balance of the gut microbiome. A clinical trial confirmed that using probiotics improved the composition of the gut microbiome, reducing cognitive impairments caused by breast cancer chemotherapy. Animal experiments also demonstrated that probiotics alleviated hippocampal damage, synaptic injury, oxidative stress, and activation of glial cells induced by chemotherapy (Juan et al., 2022). Furthermore, chemotherapy led to an increase in risk factors associated with breast cancer. Another clinical trial showed that probiotics reduced high-risk factors for breast cancer, such as chemotherapy-induced hyperlipidemia and weight gain, by modulating the gut microbiome (Juan et al., 2021). The initiation of these clinical trials indicated probiotics are becoming an adjunctive therapeutic agent for breast cancer. In addition, probiotics ameliorated symptoms of postoperative lymphedema in breast cancer patients and enhanced their immune regulation capabilities (Summer et al., 2023; Thu et al., 2023). With recent studies reporting the positive effects of probiotics on the prognosis and survival of breast cancer patients, probiotics are likely to emerge as a focal point in research on the gut microbiome and breast cancer.

In addition, several factors play crucial roles in the interaction between gut microbiota and breast cancer (Chen et al., 2022; Tian and Chen, 2024), even though these factors were not highlighted in our bibliometric analysis. For instance, epigenetic modifications are critical in regulating gene expression and maintaining cellular identity. Numerous studies have reported the interplay between epigenetics, gut microbiota, and breast cancer (Haque et al., 2022; Wu H. et al., 2023). Epigenetic modifications induce changes in oncogene expression and alter the abundance of gut microbiota. Conversely, gut microbiota produce various metabolites, particularly short-chain fatty acids (SCFAs), which inhibit histone deacetylases (HDACs; Miya et al., 2023). SCFAs have been widely reported to increase histone acetylation and alter gene expression (Mirzaei et al., 2021). Additionally, SCFAs significantly inhibit the proliferation of breast cancer cells and induce apoptosis, highlighting their potential as therapeutic agents. The study of epigenetic modifications in the context of gut microbiota and breast cancer is gaining momentum, and researchers should pay more attention to this emerging field.

### 4.4 Limitations

Similar to other bibliometric studies, this research also has some limitations. Firstly, regarding the data source, due to the limitations of bibliometric software, we were restricted to selecting a single database for analysis. In this study, we chose the Web of Science database. However, selecting a single database ensures consistency in data sources and simplifies the analysis process. Additionally, the Web of Science is a vast and comprehensive academic information resource database, renowned for its stringent selection criteria and indexing quality, which ensures that the data foundation for the analysis is reliable and authoritative. Its coverage and data quality fully meet our research needs. Moreover, bibliometric analysis provides macro-level research trends and patterns, which might not delve into the specific mechanisms and backgrounds of the research phenomena. Despite these potential flaws, bibliometric research remains the best tool for understanding scientific research trends and identifying research hotspots. Therefore, we can assert with confidence that the results obtained through bibliometric analysis are valid.

## 5 Conclusion

In this bibliometric study, we provided an overview of the distribution of articles, collaboration patterns, and trends in research hotspots within the field of gut microbiota and breast cancer over the past decade. The academic output in this field has been rapidly increasing, especially in the last 10 years, with an ever-growing number of researchers and institutions from various countries/regions joining this area of study. In recent years, employing metabolomics to study the interactions between various metabolites and the gut microbiome of breast cancer patients, as well as the use of probiotics as an adjunctive therapeutic agent for breast cancer, has become a research hotspot in this domain. The comprehensive analysis provided in this study offers new knowledge and perspectives to readers and aids researchers in identifying research hotspots. It is hoped that more research findings will be applied in clinical practice, thereby contributing to the diagnosis, treatment, and prevention of recurrence of breast cancer.

## Data availability statement

The original contributions presented in the study are included in the article/supplementary material, further inquiries can be directed to the corresponding authors.

## Author contributions

XD: Data curation, Formal analysis, Software, Visualization, Writing – original draft, Writing – review & editing. HY: Formal analysis, Writing – review & editing. LT: Data curation, Writing – review & editing. JL: Data curation, Writing – review & editing. HR: Data curation, Writing – review & editing. AG: Data curation, Writing – review & editing. LL: Writing – review & editing. HF: Writing – review & editing, Funding acquisition.
